# Posttraumatic Stress Symptoms Among Polyvictimized Women in the Colombian Armed Conflict: The Mediating Role of Social Acknowledgment

**DOI:** 10.3389/fpsyg.2021.741917

**Published:** 2021-10-05

**Authors:** José Luis González-Castro, Silvia Ubillos-Landa, Alicia Puente-Martínez, Marcela Gracia-Leiva, Gina Marcela Arias-Rodriguez, Darío Páez-Rovira

**Affiliations:** ^1^Educational Science Department, University of Burgos, Burgos, Spain; ^2^Health Science Department, University of Burgos, Burgos, Spain; ^3^Social Psychology Department, University of the Basque Country, Gipuzkoa, Spain; ^4^Catholic University of Pereira, Pereira, Colombia

**Keywords:** armed conflict, violence against women, discrimination, social acknowledgment, posttraumatic stress

## Abstract

For decades, in a situation of armed conflict in Colombia, women have suffered polyvictimization and discrimination with severe consequences that last even during the post-war peace process. This study analyzes the impact on posttraumatic stress and recovery of war-related violence against women, discrimination, and social acknowledgment. A cross-sectional study was conducted in 2019–2020. Participants were 148 women with a mean age of 47.66years (range 18–83), contacted through the NGO *Ruta Pacifica de las Mujeres* who had experienced significant personal violence. Results show that levels of perceived discrimination and lack of social acknowledgment are mediators in the relationship between polyvictimization and posttraumatic stress symptoms. Recognition by significant others, disapproval by family and the larger social milieu affects different posttraumatic stress disorder (PTSD) dimensions and therefore how these women adapt to the effects of trauma. Findings provide strong evidence that the way society and family treats women after a traumatic event affects how the victim recovers from this event. Recognition as a victim and disapproval can coexist and be a burden for women if not adequately addressed. Results stress the importance of understanding and intervening in PTSD recovery through the analysis of social processes, and not only through and individual focus.

## Introduction

Violence perpetrated during periods of armed conflict may have a lasting effect on individuals, and how they reconstruct their lives. Most studies have analyzed periods of active conflict while directing less attention toward the impact of violence during post-conflict transitional periods. Moreover, the role of gender and the effects of conflict and violence on women and their reactions to the experienced trauma have been less studied and merit a significant level of attention (Østby, 2016). In armed conflict environments, the level of all types of violence suffered by women is higher than in non-conflict-related contexts (Marsh et al., 2006). Different forms of gender violence are used as war tools both in situations of armed conflict and in post-conflict environments contributing to destabilize, humiliate, and underestimate the population while also helping foster a climate of fear and submission. Moreover, this type of gender violence acts for the perpetrators as a reaffirmation of their role in society (Manjoo and McRaith, 2011). As Álvarez-Múnera et al. (2020) have mentioned, in many instances during an armed conflict, women have had to accept the logic of a patriarchal society that has cosified their existence while at the same time suffering severe physical and psychological impacts having their voices and narratives silenced. In these post-conflict societies, women are not only the main providers in the reestablishment of social bonds and interaction processes but may also be more stigmatized than men suffering social discrimination and rejection due to the violence inflicted on them (Blay-Tofey and Lee, 2015). Moreover, because of the different types of violence they have suffered women may experience reintegration difficulties and social stigma and in many cases are ostracized by their family and community (Manjoo and McRaith, 2011).

Colombia is country which has endured six decades of internal violence and armed conflict, with over 20% of the population, mostly civilians, victims of violent episodes (Single Victims Registry (Registro Único de Víctimas), 2018). Social rejection suffered by women because of rape, forced displacement, threats, male abuse, and recruitment of their children has made their recovery more difficult and led to cycles of abuse in form of revictimization. As Kreft (2020) mentions, victims may be forced into silence by family members concerned about the negative repercussions of these experiences. Moreover, especially in cases of conflict-related sexual violence, there are also instances of impunity, victim blaming, or revictimization by the judiciary due to lack of training and gender sensitivity (Kreft, 2020) This situation can lead to a rise in social stigmatization and an absence of social support networks (Albutt et al., 2017).

In 2016, a peace accord between the Colombian government and the most relevant guerrilla (FARC) was finally struck, apparently putting an end to an armed conflict which had caused a burden to the personal, social, and economic development of Colombia (Pinzón, 2014). Feminist and pacifist social movements, such as the Ruta Pacífica de la Mujer (RPM) (Women’s Peaceful Route), have worked for over 20years to visualize the effects of war and its trauma on women’s lives empowering women in the reconstruction of their individual and collective memory and confronting the sequels derived from this violence. Nevertheless, women who have been victimized in armed conflicts are frequently overlooked in post-conflict reconstruction processes (Togeby, 1994). In fact, many of these women and associations that were mobilized both during the conflict and in the post-conflict years are now victims of new violence scenarios. This is not a situation limited to Colombia as has also been the case in countries, such as Afghanistan or Myanmar, in which persistent social structures that limit a women’s presence in civil representation contexts have not been erased after the conflict (Zulver, 2021). As Adjei (2019) pointed out gender is often used as a rhetorical device to reinforce narratives to exclude women from the peace process.

Both direct and indirect exposure to war and armed conflict have a long-lasting impact on civilian populations leading to responses, such as emotional distress or posttraumatic stress disorder (PTSD), considered to be the most relevant long-term consequence of such events (Bleich et al., 2013). Exposure to armed conflicts is related to increased anxiety, depression, and PTSD among women, in comparison with men, both during, and in post-conflict situations (Rugema et al., 2015; Charlson et al., 2019). Mundy et al. (2020) studying refugees in Denmark indicated that the number of traumatic events experienced by these participants had an influence on symptoms, such as depression, anxiety, and somatization to a greater extent in women than in men. In this same study, trauma burden and PTSD symptoms were moderately correlated for women but not for men. Various studies have found that exposure to multiple traumatic or life-threatening events, in comparison with singular events, has a greater negative impact and explains more variance in PTSD symptoms (Green et al., 2000; Breslau et al., 2008; Ogle et al., 2014).

A conflict that heavily impacts a society’s social system, such as an armed conflict or persistent low intensity war, affects not only an individual but also the whole social fabric. As such, recovering from trauma caused by suffering violence in these contexts should not only be analyzed as an individual phenomenon. As Charuvastra and Cloitre (2008) mention, posttraumatic stress recovery is highly dependent on social phenomena because social experiences, both positive and negative, play a pivotal role in the way people respond to traumatic events. An analysis on the impact of the social and relational framework in which trauma responses occur is needed to achieve a more thorough understanding of trauma stress and its recovery, not only analyzing the event from a mere individualistic perspective (Maercker and Horn, 2013; Woodhouse et al., 2018).

Meta-analytic reviews have shown that perceived discrimination has negative consequences on an individual’s mental health (Schmitt et al., 2014). Nevertheless, fewer studies have examined the association between discrimination and PTSD, although the latter is related to poor quality of life and mental and physical health (Brooks Holliday et al., 2020). Verelst et al. (2014) found that discrimination significantly explained the impact of war-related sexual violence in Eastern Congo adolescents and PTSD. A study analyzing this association with war victims conducted by Ibrahim et al. (2018) found in a sample of Yazidi women victim of enslavement and genocide that the relationship between trauma events and depression was mediated by perceived social rejection. Nevertheless, the mediation effect for PTSD was non-significant.

As PTSD is related to social responses toward a victim, social acknowledgment – defined as referring to how appreciated, as a victim of a traumatic event, an individual feels he or she is by family and the general population is an important factor in the psychological adaptation to the traumatic stressor and future recovery (Maercker and Müller, 2004).

Maercker and Horn (2013) have shown that interpersonal traumatic events relate to high levels of PTSD through the social process of social acknowledgment. Woodhouse et al. (2018) conclude that social acknowledgment may have a stronger predictive power than social support in a posttraumatic environment.

**Figure 1:**
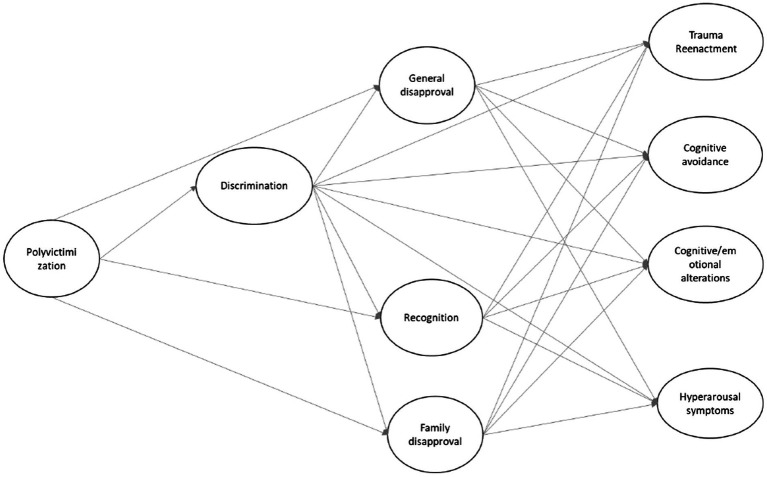
Theoretical integrated model depicting observed paths among study variables.

This article further elaborates on the previously mentioned literature developing and testing a social model of trauma symptoms as shown in Figure 1. The complete model is exploratory because the combination of variables has not been fully tested before. The proposed model includes social processes (discrimination and social acknowledgment) that may lead to the preservation of the negative effect that women’s polyvictimization has on PTSD. The structure of the model and the order of the variables reflect past theory and empirical evidence, as mentioned before (i.e., the relationship between suffered traumas and social processes or between social acknowledgment and PTSD). Due to the nature of the study, instead of inferring a causal direction, we will establish relations between constructs. The effect of personal trauma (polyvictimization) on PTSD is predominantly explained through the judgments (discrimination and social acknowledgment) that the members of a society make regarding the experienced trauma. Previous literature (Kohli et al., 2014; Ibrahim et al., 2018) regarding social acknowledgment and rejection suggests that an individual’s social network, family, and society at large respond to trauma that has afflicted a person and that a victimized woman may perceive this response in terms of more or less discrimination and social acknowledgment. Some of the indirect effects of trauma on PTSD will be mediated by these social processes. A woman in these contexts of armed conflict who has experienced different types of personal trauma (psychological, physical, or sexual violence) may suffer more discrimination, and less social recognition, from their family, community, and society in general (Manjoo and McRaith, 2011). In this case, the individual perception of social acknowledgment will be low because discrimination hinders the possibility of sharing sympathy and recognition of the experienced traumas. Based on empirical data on social acknowledgment, lower levels of this type of recognition will be linked to more posttraumatic stress symptoms (Maercker and Horn, 2013).

The objective of this research is to test a social model of trauma to explain the variance in PTSD symptoms. This study will analyze how experienced violence, and the number of violent acts one has suffered (polyvictimization), affects a group of women’s posttraumatic stress and recovery. This relationship will be mediated both by the levels of perceived discrimination a person has experienced and the degree in which not only individuals, but also social groups influence the person by judging the victim’s unique state and acknowledging, or not, the victim’s current difficult and/or traumatic situation.

## Materials and Methods

### Sample

The sample comprised 148 women contacted through the NGO RPM. RPM is a women’s social movement that has been directly involved for over 25years in making the impacts of war on women’s lives and bodies visible and trying to influence the process, negotiation, and non-violent resolution of conflicts. The 25-year experience of the RPM has generated in many women, victims of the conflict, enough confidence and legitimacy to approach, get involved, and become part of this organizational process. Due to the nature of the NGO and the aims of this study, all the women who voluntarily participated in the study throughout their lives had experienced victimizing events (e.g., forced displacement, forced recruitment of their children, sexual violence, stigmatization, murder of family members and loved ones, massacres, and harassment, among other victimizing events) that in many cases, they did not recognize as such for a long time. However, their membership in the RPM has allowed them to transform and reconsider these personal and social experiences of violence. For many women, the RPM is the only resource they have to recognize and highlight in a safe context all the suffering they have experienced with no judgments made and in which the center of the narrative is a women’s life and experience. The exclusion criteria were as: a) being a minor because research processes with minors require other protocols and authorization from adults that are difficult to obtain; b) women who had participated in the RPM for a period of less than 2years because these women may have fewer resources to face questions about reenacting very painful experiences. Women who have been engaged in some way with the RPM for over 2years probably have more resources to cope with the questions that bring up painful memories without stressing revictimization.

The participating women belonged to the areas of Bolívar., Santander, and the Eje Cafetero. The regions and municipalities where the study was carried out were selected for two main reasons: (1) the RPM has recognition and legitimacy among women victims in the regions where the study was carried out, which facilitated the meeting and their willingness to participate in the study; (2) selected regions, such as Bolivar and Santander, have had a history of violence due to the armed conflict that is socially recognized in the country. Also, in the Eje Cafetero region, violence due to the armed conflict has been invisibilized and minimized. Therefore, we intend with this study to contribute to its visibility and recognition.

Mean age of the participants was 47.66years (*SD*=15.65) ranging from 18 to 83years. Table 1 outlines the population demographics of the participating women.

**Table 1:** Descriptive analyses. Socio-demographic characteristics.

	N (range)	%
*Region*
Bolívar (Cartagena, Aragón and San José del Playón)	31	20.9
Eje Cafetero (Caldas and Risaralda)	97	65.6
Santander	9	6.1
Missing	11	7.4
*Race*
Afro-Colombians	32	21.6
Mixed	18	12.2
Native	97	65.5
Missing	1	0.7
*Residence*
City	71	48
Village	76	51.3
Missing	1	0.7
*Labor activity*
Working	36	24.3
Unemployment	3	2
Student	19	12.9
Housewife	77	52
Retired	8	5.4
Others	5	3.4
*Education*
None	37	25
Primary School	42	28.4
Secondary school	52	35.1
University	17	11.5
*Marital status*
Single	46	31.1
Married	43	29.1
Consensual union/ Romantic relationship	22	14.8
Widow	20	13.5
Divorced	16	10.8
Missing	1	0.7
*Religion*
Catholic	127	85.8
Others	21	14.2
*War-related violence (Yes)*
Physical	82	55.4
Psychological	144	97.3
Sexual	34	23
*Forced displacement (Yes)*	85	57.4

An aggregate score of accumulated trauma (polyvictimization) indicated that *n*=62 women (41.9%) had suffered only a single form of violence: physical, psychological, or sexual; *n*=60 (40.5%) two of them; and *n*=26 (17.6%) all three types of violence.

### Procedure

This is a cross-sectional study conducted in Colombia in 2019–2020. In order to engage participants in the study, first, one of the researchers in Colombia, who is an active member of the RPM for 10 years, contacted the RPM coordinators in the different regions where the NGO has offices. Second, various coordination meetings were held between the research team and RPM representatives to explain the aim and procedure of the research. Third, potential participants were contacted and recruited for the study (by telephone) through the RPM coordinators. Four, meetings of the coordinators with the participating women were held in each of their territories to explain the research project and to set the dates for data collection. Finally, the researchers from Colombia traveled to the territories to ensure the participation of the women without additional effort for them, since many of them live in very isolated rural areas and other are leaders who have been threatened and silenced and the only resource they have to explain their experience is that provided by the RPM. The data collection was carried out in their own personal spaces, such as booths, the offices of partner organizations, and rooms that had optimal conditions (light, ventilation, chairs, and tables) for carrying out the procedure.

RPM experts worked with one of the researchers to carry out the data collection in the municipalities of Supía and Pereira (Eje Cafetero), Cartagena, Aragón and San José del Playón (Bolívar) and Santander. Approval for the study was given by the Ethical Committees of the institutions involved as: Burgos University and Basque Country University. Once the informed consent forms had been signed, respecting the ethical principles established in the Declaration of Helsinki, the evaluation protocol was completed in groups of three or four women under professional supervision. The protocol took approximately 90min to complete.

### Instruments

#### Violence Scale

Based on the systematic review conducted by Ba and Bhopal (2017), an *ad hoc* scale was developed to address information on three types of violence. This review is one of the most recent, specific, and comprehensive in analyzing different types of violence in an armed conflict zone. Eleven forms of sexual violence (e.g., rape, forced marriage, and sexual slavery), 11 categories of physical violence (e.g., being pushed and/or grabbed), and 10 types of psychological violence (e.g., being harassed and threatened) were identified. The response option was dichotomous (yes/no). An aggregated violence score (polyvictimization) was created to include those women who had suffered one, two, or all three types of violence.

#### Everyday Discrimination Scale

The original scale was developed by Williams et al. (1997). In this study, an Verelst et al. (2014) for girls who have suffered violence in a context of war was applied. The (Everyday Discrimination Scale) EDS in general assesses the underlying construct of perceived discrimination equivalently across diverse racial/ethnic groups. It is one of the most widely used discrimination scales in epidemiologic and public health research. The 14 items (yes=1/no=0) focused on experiences of different aspects of stigmatization as a result of violence, including perceived discrimination and social exclusion in the family and community context (e.g., being treated as if you were different, being isolated by the nuclear family, and being treated badly by family members, people act as if they are better people than you). The total score was calculated with the sum of items. The higher the score the greater the perceived discrimination.

#### Social Acknowledgment Questionnaire

This scale (Maercker and Müller, 2004) has been widely used, with good psychometric properties in women samples and accurately assesses the degree to which people feel validated and supported by their social environment after a traumatic event. This is a self-report scale composed by 16 items measuring social acknowledgment as a victim or survivor. It is rated on a Likert scale from 0 (not at all) to 5 (completely). It contains the following subscales: recognition as a victim or survivor (six items, “My friends feel sympathy for what happened to me”) (0–30), general disapproval (five items, “Most people cannot understand what I went through”) (0–25), and family disapproval (five items, “My experiences are underestimated in my family”) (0–25). These subscales capture information on social acknowledgment in three contexts: family, friends, and people in the community. The higher the score the greater the perceived social acknowledgment as a victim and greater general and family disapproval.

#### Posttraumatic Stress Disorder Symptom Severity Scale (EGS-R)

This scale (Echeburúa et al., 2016) evaluates the severity of posttraumatic stress symptoms. It is an instrument that has been recently created in the Spanish language and shows good psychometric properties. It comprises 21 items (e.g., having unpleasant and recurring dreams about the event) and consists of four scales based on the DSM-5 diagnostic criteria assessing a) trauma reenactment (five items, e.g., “do you experience involuntary unpleasant and repetitive memories or images of the event), b) cognitive avoidance (three items, e.g., “do you avoid or make efforts to take your mind off memories, thoughts or feelings linked to the event because they cause emotional displeasure?”), c) cognitive/emotional alterations (seven items, e.g., “do you experience a constant negative mood in the form of terror, anger, blame or shame?”), and d) hyperarousal symptoms (six items, e.g., “Are you in a constant state of alarm, for instance suddenly stopping to see who’s next to you. etc. since the event took place”). Responses are given on a Likert-type scale in accordance with the frequency of the symptoms (from 0=never to 3=5 or more times a week/very often). Total scores on the global scale range from 0 to 63.

All scales, except the EGS-R, have been translated into the Spanish language following the International Test Commission (2017) recommendations. These scales were first translated from English to Spanish by a bilingual speaker. They were then revised by two members of the research team to analyze if the translation captured the meaning of the original items. Consequently, they were back translated into English by a native speaker to correct any mistakes or omissions. Finally, they were adapted into Spanish used in Colombia and a pilot study with five participants was performed to check if the scales were correctly understood.

### Data Analysis

A series of descriptive statistics were conducted (means, standard deviations, frequencies, and percentages) to describe the sample and variables under study. Cronbach’s alpha was used to analyze reliability of the instruments. Multivariate analysis and Pearson correlation coefficients between variables included in the study were calculated. Statistical analyses were performed using SPSS v.25. Effect sizes of the mean differences were estimated using Cohen (1988) criteria. A small effect was conceptualized as *d*=0.20, medium *d*=0.50, and large *d*=0.80.

The impact of armed conflict-related violence against women, discrimination, and social acknowledgment of posttraumatic stress was analyzed by a path analysis. These path analyses with observed variables were performed on raw data files using the maximum likelihood estimation procedure in the Mplus v.8.5 software (Muthén and Muthén, 1998-2007).

A series of global fit indices were used to determine whether the data fitted the proposed path model, including a chi-square test of model fit (*χ*^2^), the root mean square error of approximation value should be less than 0.08 to declare satisfactory fit, the comparative fit index value should be greater than 0.90, the Tucker Lewis index value should be greater than 0.90, and the standardized root mean square residual should be less than 0.05 (Kline, 2010). Indirect effects were calculated using 10,000 bootstrapping samples. A conditional indirect effect is considered statistically significant if the confidence interval (CI at 95%) does not include the value 0.

## Results

### Multivariate and Correlation Analyses

Differences between women who had suffered one, two, or all types of violence (physical, psychological, and sexual) were found for discrimination (one type: *M*=2.41, *SD*=2.44; two types: *M*=2.65, *SD*=2.84; three types of violence: *M*=5.34, *SD*=4.52, *F*=9.18, *p*=0.0001, *d*=0.65) and recognition as a victim (one type: *M*=9.54, *SD*=7.30; two types: *M*=6.08, *SD*=6.07; three types of violence: *M*=8.46, *SD*=7.99, *F*=3.86, *p*=0.023, *d*=0.44). Post-hoc test showed that victims of physical, psychological, and sexual violence were more discriminated than women who suffered two or one type of violence. Furthermore, women who suffer two types of violence were less recognized as victims than women who suffered one type of violence. Results also showed more discrimination (yes: *n*=34, *M*=4.47, *SD*=4.38, no: *n*=114, *M*=2.59, *SD*=2.66, *F*=9.38, *p*=0.003, *d*=0.60) and family disapproval (yes: *M*=10.71, *SD*=7.43, no: *M*=8.84, *SD*=5.66, *F*=5.81, *p*=0.017, *d*=0.30) in women who suffered sexual violence than among non-victims of this type of violence. Discrimination was also more common among victims of physical violence than among non-victims (yes: *n*=82, *M*=3.54, *SD*=3.66, no: *n*=66, *M*=2.39, *SD*=2.45, *F*=4.72, *p*=0.031, *d*=0.36). There were non-significant differences between victims and non-victims of psychological violence in the variables under study.

In Table 2, we present the Cronbach’ alphas of the scales and a series of correlations to explore the relations among variables. Polyvictimization was positively associated with discrimination but also with more family disapproval, indicating that both social and family rejection increased with the accumulated trauma experiences. It was also linked positively to the trauma reenactment symptom of PTSD. Furthermore, the EDS total score was positively related to general and family disapproval and with recognition as a victim. Moreover, it was also positively associated with trauma reenactment, hyperarousal, and total EGS-R score. Recognition as a victim correlated positively with general disapproval and negatively with family disapproval. It was also positively related to trauma reenactment. General and family disapproval also correlated significantly. General disapproval was related positively with all the EGS-R dimensions. Family disapproval is also linked with more cognitive and emotional alterations, hyperarousal, and the total EGS-R score.

**Table 2:** Correlation Analyses.

	*α*	*M*	*SD*	1	2	3	4	5	6	7	8	9
Polyvictimization	0.85			1								
Discrimination-EDS	0.85	3.03	3.22	0.45^∗∗∗^	1							
General disapproval-SAQ	0.82	9.27	6.13	0.15	0.30^∗∗∗^	1						
Recognition as a victim-SAQ	0.80	7.95	7.09	−0.10	0.25^∗∗^	0.21^∗^	1					
Family disapproval-SAQ	0.66	10.11	4.39	0.23^∗∗^	0.37^∗∗∗^	0.23^∗∗^	−0.26^∗∗^	1				
Trauma reenactment-EGS-R	0.95	8.41	5.54	0.17^∗^	0.16^∗^	0.60^∗∗∗^	0.19^∗^	0.15^†^	1			
Cognitive avoidance-EGS-R	0.86	5.25	3.32	.15^†^	0.10	0.48^∗∗∗^	0.03	0.08	0.81^∗∗∗^	1		
Cognitive and emotional alterations-EGS-R	0.89	8.69	6.51	0.11	0.15	0.53^∗∗∗^	−0.12	0.35^∗∗∗^	0.77^∗∗∗^	0.72^∗∗∗^	1	
Hyperarousal-EGS-R	0.86	7.26	5.40	0.10	0.26^∗∗^	0.53^∗∗∗^	0.04	0.25^∗∗^	0.81^∗∗∗^	0.73^∗∗∗^	0.83^∗∗∗^	1
Total EGS.R	0.96	29.61	19.02	0.14	0.19^∗^	0.59^∗∗∗^	0.03	0.25^∗∗^	0.92^∗∗∗^	0.86^∗∗∗^	0.92^∗∗∗^	0.93^∗∗∗^

*N*=148. EDS, Everyday Discrimination Scale; SAQ, Social Acknowledgment Questionnaire; and EGS-R, Impact of Event Scale-Revised.

∗∗∗ *p*≤0.01;

∗∗ *p*≤0.01;

∗ *p*≤0.05;

† *p*<0.10.

### Paths From Polyvictimization to PTSD Symptomatology

**Figure 2:**
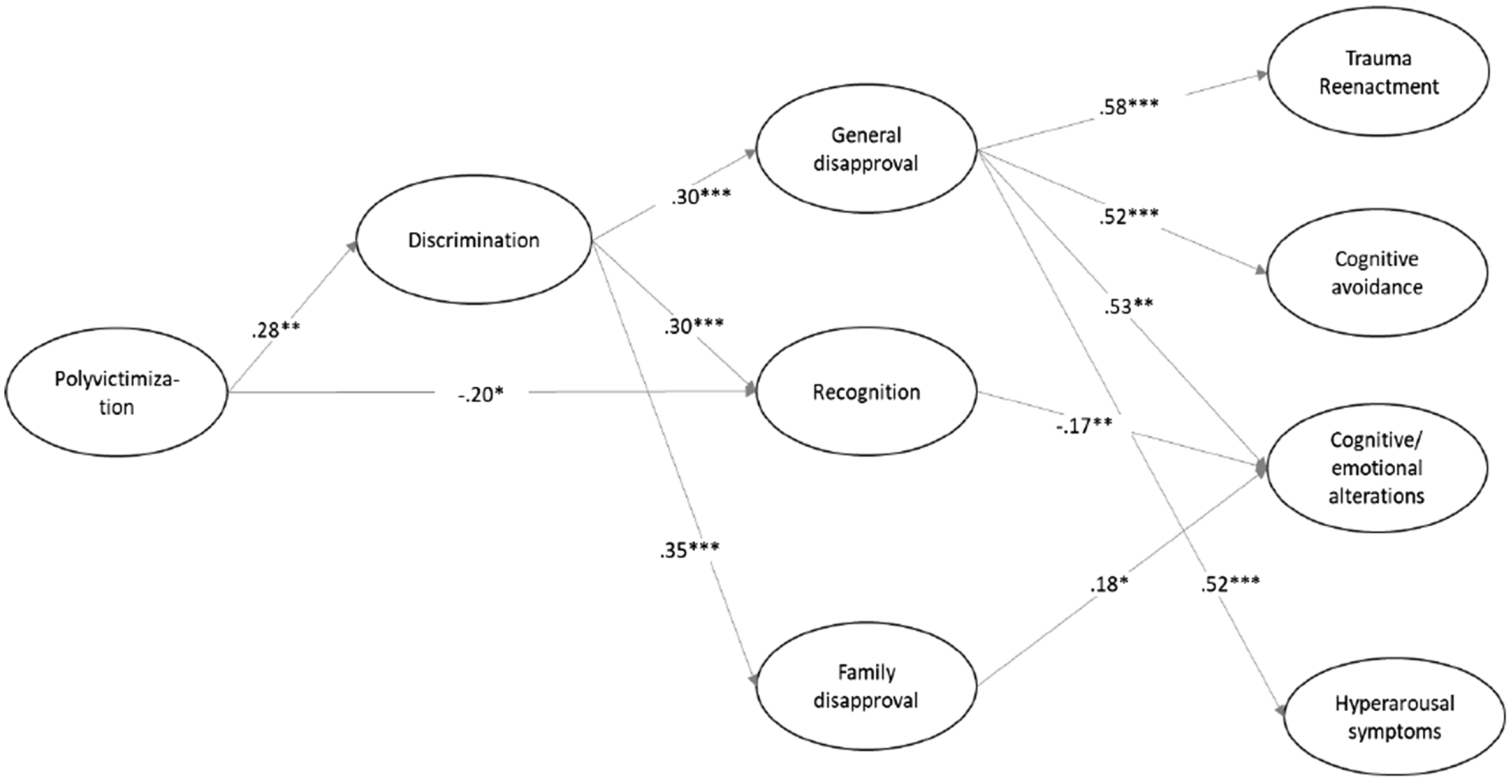
Path analysis and standardized coefficients. Non-significant coefficient were not shown (*p*≤0.50) Model fit: *χ*^2^ (*df*=0.36, *N*=148) = 746.44, *p* = 0.0001, CFI=0.99, TLI=0.96, RMSEA=0.07, SMR=0.014, 95% CI [0.001 0.153].

We hypothesized a PTSD model that yielded an adequate fit to the data (see Figure 2). Results revealed that polyvictimization is positively related to discrimination. Moreover, discrimination had a significant direct effect on general and family disapproval, and on recognition of others as victim. The indirect effects were significant in general (*β*=0.09, *Se*=0.03, *p*=0.018) and family disapproval (*β*=0.10, *Se*: 0.04, *p*=0.018), and recognition as a victim (*β*=0.08, *Se*=0.03, *p*=0.005) indicating that discrimination mediates the relationship between polyvictimization and the different dimensions of social acknowledgment as a victim. Discrimination increases the effect of being a victim of more than one type of violence on social and family disapproval and on being recognized as a victim. Nevertheless, discrimination has no direct effect on the PTSD’ dimensions.

In a second step, the direct effect of social acknowledgment on PTSD and the indirect effect of discrimination on the four dimensions of PTSD through general disapproval, family disapproval, and recognition as a victim were tested.

First, results indicated that general disapproval had a direct and positive effect on trauma reenactment, cognitive avoidance, cognitive and emotional alterations, and hyperarousal. The indirect paths were significant from discrimination to trauma reenactment (*β*=0.18, *Se*=0.05, *p*=0.0001), cognitive avoidance (*β*=0.16, *Se*=0.05, *p*=0.0001), cognitive and emotional alterations (*β*=0.16, *Se*=0.05, *p*=0.0001), and hyperarousal (*β*=0.15, *Se*=0.04, *p*=0.0001) through general disapproval.

Second, the direct path from family disapproval to cognitive and emotional alterations was also significant. Moreover, the indirect effect showed that family disapproval explained the emotional and cognitive alterations caused by social discrimination (*β*=0.07, *Se*=0.03, *p*=0.050).

Third, recognition as a survivor was negatively related to cognitive and emotional alterations and the indirect effect indicates that this recognition decreases the effects of discrimination on this PTSD symptom (*β*=−0.05, *Se*=0.02, *p*=0.022) (see Figure 2).

## Discussion

The way society treats a person after a traumatic event affects how the victim recovers from this event as well as reflecting the social fabric of communal relationships. Results from this study show that social acknowledgment has an impact on how women who have survived violence in an armed conflict process their traumatic experiences emotional and cognitively. Polyvictimization and discrimination affect PTSD symptoms through how the rest of the community accepts or rejects victims. Recognition or disapproval by family, significant others, and the larger social milieu has an important, and differential, effect on these women’s psychological adaptation to the consequences of trauma (Maercker and Müller, 2004; Mueller et al., 2008).

The dose–response relationship literature, and more specifically in the case of Colombia, results from Campo-Arias et al.’s (2017) research show that polyvictimization was related to more discrimination in comparison with victims of a single event and that discrimination is a form of indirect violence (Fernández Cediel, 2014). Moreover, polyvictimized women have suffered the stigma-discrimination secondary complex related to the traumatic event (Campo-Arias and Herazo, 2015). Victims of sexual violence (Rees et al., 2011) and forced displacements (Burgess and Fonseca, 2020) in situations of armed conflict show how discrimination and stigma may have as severe an impact as the experienced trauma. This not only represents another potential stressor for victimized women, but also multiplies the probability of experiencing PTSD symptoms after suffering a traumatic event (Kennedy et al., 2014) and is one of the main barriers impeding participation in actions that try to change one’s life (Burgess and Fonseca, 2020). Most of the women in this study are members of vulnerable social groups in society with limited access to critical social networks that could offer opportunities for a positive change and mental health recovery.

Maercker and Horn (2013) have shown that low levels of social acknowledgment derived in higher levels of PTSD cognitions through strengthening feelings of fear and mistrust. Rebolledo and Rondón (2010) stress the importance of social approval or rejection and are critical of a medical and individualistic model of PTSD that does not consider the social and political context in which women live. To reduce posttraumatic stress, it is as important to work on the symptoms, as it is to understand and try to change the context in which these violent acts take place. Although individual interventions may mitigate the impact of being a victim of violence, these women in many cases must return to a social and relational context (with family and friends) in which it is necessary to integrate the process of collective recovery. Public recognition, or in other words, the transit from a private (suffering) to a public sphere helps restore that which was sullied by trauma. The use of violence in armed conflicts and the persistence of these acts, including by civilians, in post-conflict environments clearly destroys the social fabric of families and communities. As a result, there is an all over impact on the health of individual victims (Verelst et al., 2014).

This study shows that society, close relations, or one’s own family disapproval or recognition (social acknowledgment) increases the negative effect of both discrimination and polyvictimization on a victim’s PTSD symptoms. Moreover, although a victim may receive recognition as such by one’s close social group, at the same time, they may face general and family disapproval. In Colombia during the last few years, there have been developments favoring a holistic reparation of the violence suffered by women mainly through the actions and interventions of social movements (Fernández Cediel, 2014). Women from this study are part, or have ties, with the RPM, an NGO in favor of reparation and a negotiated end to the armed conflict in Colombia. It is reasonable to argue that these polyvictimized and discriminated women have a more positive environment in which they are acknowledged as victims by those significant within their context (friends, neighbors, local authorities, etc.). These women have witnessed how through the actions of associations, such as the one they belong to, the Colombian government has implemented different laws supporting women victims of violence (e.g., law 1,257 from 2008 or resolution 1,441 in 2013).

Nevertheless, these dispositions have not necessarily been implemented by successive governments. There are still flaws in the support local and national government departments offer women victims of armed conflict violence. This situation does not allow for significant changes in the role and image of women in society and could explain why the more discrimination women victim of armed conflict suffer, the higher both general and family disapproval (Casa de la Mujer and Ruta Pacífica de las Mujeres, 2010).

Disapproval and recognition not only play a different role in linking polyvictimization and discrimination to PTSD, but family and general disapproval also impact on different PTSD symptoms. General disapproval, the opinion of an abstract set of society members, leads to an increase in the effect of discrimination in all symptoms of posttraumatic stress. That is, a recrudescence of the re-experience of the traumatic event that induces a state of high anxiety, cognitive avoidance, emotional reactivity, and hyperarousal. These findings support the idea that social reactions of disapproval and lack of support are related to PTSD in women survivors. Moreover, these results are consistent with other studies that have found similar results showing that general disapproval was the only factor of the Social Acknowledgment Questionnaire (SAQ) that in different contexts was positively and significantly related to PTSD (Koenen et al., 2003; Mueller et al., 2008; Schumm et al., 2014; Lis-Turlejska et al., 2018). Xu et al. (2016) state that a positive change in social recognition was linked with a decrease in PTSD after treatment. These results support the idea that social disapproval may be especially psychologically harmful for survivors trying to recover from these events (Fontana et al., 1997; Koenen et al., 2003). Moreover, Ullman (2010) stresses that the different sources of recognition may be more or less influential depending on, for instance, cultural factors. Maercker et al. (2009) found that traditional values as those predominant in cultures, such as Colombia (Casas and Méndez, 2019), inhibit social recognition as a victim. In this case, a low sense of social acknowledgment was accompanied by the need to tell others about the trauma and led to more PTSD. Our results suggest that general disapproval is most relevant in its capacity to explain PTSD results. As a result, it should be considered an important aim in interventions whose objective is to promote trauma recovery and reduce PTSD symptomatology.

On the other hand, both family disapproval and recognition as a victim by significant others mediate the effect of discrimination on the cognitive and emotional PTSD problems. The way disapproval and recognition affect PTSD seems to be completely different: Family disapproval leads to an increase in the cognitive and emotional symptoms while recognition decreases the effect of discrimination and polyvictimization. In a family, its members may suffer different impacts which combined erode family interactions and force the group to reconstruct itself. The social effects of armed conflict not only affect individuals but also the social structure of close kin groups. In many cases, the family milieu has turned into something alien which is no longer a reference for security and identity. Although many families try to reorganize their relationships to continue to support its members, in certain cases, and due to the sequelae of the traumatic situation, we find that blame, rage, hurt, and impotence emerge in family interactions. This is especially the case in victims of sexual violence who suffered more discrimination and family disapproval than those non-victims of such type of violence. This family conflict in situations of armed conflict is sometimes resolved through networks of community sociability.

Moreover, as Jones et al. (2006) have shown, the positive effect of recognition as a victim by significant others has less impact than the negative effect of general disapproval. The political and social context of Colombia may also limit the effect of recognition by significant others. In countries in which armed conflict has persisted for decades, previous periods of unpunished violence have led to a continuing acceptance of violence against socially and politically marginalized groups, such as is the case of women (Tarnaala, 2019). Different reports have shown that if indiscriminate violence against women was common during the conflict, targeted assassinations and threats turned into common practice after the demobilization. Moreover, this violence in numerous cases is directed toward social movements, such as the RPM in Colombia, who are part of the scarce social support networks available to these women in fractured societies. Surveys conducted in different areas of the world have revealed that people who have a more active political and civic interest have more possibilities of being a victim of different types of violence (Bateson, 2012). In the case of women’s organizations and individual activists, murders, attacks, and direct threats were a warning to other women to abandon social activism and “return” to more traditional gender roles (Defensoría del Pueblo, 2014). To make matters worse, after the peace accords and perpetrators, people who have killed, mutilated, and raped, often returned to live in the same communities in which their victims and families, were living. Currently, crimes against women are committed with the same unaccountability and impunity as during the conflict. These women’s lives are still now on an organizational, political, and socioeconomic level restricted and under control. Notwithstanding these limitations, in many cases, only women survivors and their organizations are able to preserve the memory of the repression, organize public events of solidarity with victims, and forced the government to play an active role in their protection (Tarnaala, 2019).

We have seen that polyvictimization suffered by women in a context of armed conflict has two distinct phases. First is the instance in which the violent acts took place. Second, we find the moment of impunity and lack of mechanisms to recognize what has happened, a point in which these acts are “quietly” forgotten. This phase can lead to irreparable repercussions in victims. These are two complementary forms of psychological damage.

### Practical Implications

This study supports placing a stronger emphasis on applying social psychological theories and constructs to the study of traumatic events and their impact on mental health. These results may have important clinical implications in interventions with women victims of violence in armed conflict and post-conflict situations. It is necessary to develop a holistic perspective that includes both the victim and their social environment (family, community, and society at large) (Kelly et al., 2012).

Moreover, the United Nations’ Security Council Resolution 1,325 not only addressed the issue of gender inequalities in conflicts, and how it disproportionally affects women, but also stressed the need to include gender perspective in peace conflict resolution mechanisms. Women are not only victims of armed conflicts, but also leaders and peacemakers. This implies not reducing the role of women to more traditional spheres of peace education but engaging in women peacemaking, peacekeeping, and peacebuilding activities, although everyday interventions show us that women still do not systematically participate in decision making about reconstruction and peace negotiations (Jansen, 2006; Adjei, 2019). This aim must be achieved through the direct involvement of women, and women’s organizations, victim of gender violence in armed conflict situations, but also through counseling and support from professionals well versed on the nuances of each context and situation. For instance, Jansen (2006) stresses how social workers, and other health and social services professionals, are trained to understand not only trauma suffering but also resilience and a person and community’s coping mechanisms. NGO’s, governments, and professional health care providers must learn to correctly assess needs and understand how the larger interplay of social relations and culture may resound on the impact of trauma on individuals and groups, mobilizing women to participate in effective social reconstruction. Women and their families should be offered services, resources, and means to facilitate a group/social trauma approach and provide positive psychosocial support that does not exacerbate trauma or discrimination but rather individual and group integration, recovery, and resilience.

There is a need to develop interventions in women’s most proximal social environments and promote family therapy and community interventions to strengthen a victim’s closest social networks (Verelst et al., 2014). Nevertheless, interventions based on social capital must be socially and politically adapted in order to increase their efficacy. The interdependence of the various community intra- and inter-social contexts will assign a different weight, influence, and importance to the improvement of a victim’s mental health in each of these contexts (Flores et al., 2018). The results from this study stress that a collective social change could be the most appropriate proposal to address the mental health needs of women victims of armed conflict. Since stigmatization and general disapproval explain a large part of the impact of violence on the mental health of women victims, interventions should include approaches that take into consideration the social system. Stigma and disapproval toward women are enhanced by mass media messages, the initiative of government representatives and the actions, and omissions, of many members of the general public. More awareness interventions are needed to alert the general population, police, and army of the need to create safe havens that have banned stigma and discrimination directed toward these severe violations of human dignity and rights. Promoting the social approval of victims would be beneficial in family and community environments weakened by prolonged armed conflict. The aim would be to improve their ability to take actions and strengthen the positive result which a shared acknowledgment by those social groups closer to the victim may have on survivors’ mental health (Campbell, 2019). An approach that integrates more individualistic clinical psychological perspectives, and more collective sociopolitical variables offer an opportunity to promote long-lasting changes in more vulnerable communities, such as those from which most of the women who participated in this study come from Burgess and Fonseca (2020).

### Limitations

The number of women who participated in the research was low, although the complexity of the questionnaire, and the physical difficulty of accessing the terrain and sites did not allow for a larger sample which would be necessary to explore the mediating effects of social acknowledgment found in this study. The cross-sectional design of the study does not allow for an analysis of causation. To address this problem, more longitudinal studies should be designed and implemented in the future.

The nature of the participants and their implication in the actions of an NGO does not allow for a generalization of results to other women who have suffered the same violence but who may lack the assistance of a structured organizational network in which to share one’s experiences. Moreover, participants answered a self-report remembering their actions and feelings years after the events took place in many cases. This may pose a problem of recall and memory reconstruction, although due to the nature of the events, these studies will always be reconstructive since objective data from the moment the events took place, and women suffered these episodes of violence, are very difficult to obtain.

## Conclusion

Polyvictimization and posttraumatic stress symptoms are related indirectly through discrimination and lack of social acknowledgment. Results of the study stress the importance of understanding and intervening in PTSD recovery through the analysis of social processes and not only as an individual variable. Recognition as a victim and disapproval can coexist and be a burden for women if not adequately addressed. Family, close relations, and the more general society have different impacts on the various symptoms of PTSD, and as such must be considered when intervening and designing programs for women victims of armed conflict induced violence. Enhancing the recognition of women who have suffered violence by significant others and decreasing family and social disapproval can help recover women’s mental health, despite the trauma caused by violence.

## Data Availability Statement

The raw data supporting the conclusions of this article will be made available by the authors, without undue reservation.

## Ethics Statement

The studies involving human participants were reviewed and approved by Ethical Committees of the institutions involved: Burgos University and Basque Country University. The patients/participants provided their written informed consent to participate in this study.

## Author Contributions

SU, AP, and JL contributed to the conception and design of the study. GM and AP collected the data. MG and AP organized the database. JL, SU, and AP performed the statistical analysis. JL, SU, AP, DP, and MG wrote the first draft of the manuscript. All authors contributed to the manuscript revision, reading, and approving the submitted version.

## Funding

This work was supported by the grants from the Spanish Ministry of Science and Innovation (PID2020-116658GB-100) (PSI2017-84145-P) and (PID2020-115738GB-I00). Research group grant from the Basque Government (‘Culture, Cognition, and Emotion’ Consolidated Group; IT1187-19), the Development Cooperation Office of the Basque Country University, and grant 2019/00184/001 awarded by the Junta of Castilla y León (Spain) to the Social Inclusion and Quality of Life (SIQoL) research group.

## Conflict of Interest

The authors declare that the research was conducted in the absence of any commercial or financial relationships that could be construed as a potential conflict of interest.

## Publisher’s Note

All claims expressed in this article are solely those of the authors and do not necessarily represent those of their affiliated organizations, or those of the publisher, the editors and the reviewers. Any product that may be evaluated in this article, or claim that may be made by its manufacturer, is not guaranteed or endorsed by the publisher.
